# The Feasibility and Acceptability of Culturally Appropriate Pulmonary Rehabilitation for Adults with Chronic Obstructive Pulmonary Disease in Sri Lanka: Randomized Controlled Trial

**DOI:** 10.2147/COPD.S527013

**Published:** 2026-04-30

**Authors:** Akila R Jayamaha, Chamilya H Perera, Mark W Orme, Ravini De S Karunatillake, Amitha Fernando, Andy Barton, Michael C Steiner, Jesse Matheson, James Manifield, Amy C Barradell, Suresh Chathurantha, Ruwin R Dias, Thamara D Amarasekara, Savithri W Wimalasekera, Sally J Singh

**Affiliations:** 1 Faculty of Nursing, KIU, Colombo, Sri Lanka; 2 Department of Respiratory Sciences, University of Leicester, Leicester, UK; 3 Centre for Exercise and Rehabilitation Science, University Hospitals of Leicester NHS Trust, Leicester, UK; 4 Central Chest Clinic, National Hospital of Sri Lanka, Colombo, Sri Lanka; 5 Department of Economics, University of Sheffield, Sheffield, UK; 6 Faculty of Music, University of Visual and Performing Arts, Colombo, Sri Lanka; 7 Faculty of Allied Health Sciences, University of Sri Jayewardenepura, Nugegoda, Sri Lanka; 8 Faculty of Medical Sciences, University of Sri Jayewardenepura, Nugegoda, Sri Lanka

**Keywords:** COPD, culturally adapted PR, Colombo Sri Lanka, practicality, suitability

## Abstract

**Purpose:**

Pulmonary rehabilitation (PR) is recommended internationally for individuals with chronic obstructive pulmonary disease (COPD), but there is limited evidence and practice of PR in Sri Lanka. Key challenges for PR such as poor accessibility, uptake and completion need to be addressed when designing and delivering new PR programmes. Accordingly, this study determined the feasibility and acceptability of culturally adapted PR for adults with COPD in Sri Lanka.

**Patients and Methods:**

A randomized controlled feasibility trial was conducted with 50 adults living with COPD in Colombo, Sri Lanka. A culturally adapted PR comprised a 6-week rolling programme with sessions conducted twice every week. Sessions involved endurance and resistance exercise training, education and cultural adaptations of nutritional support and group singing. The control group received usual care, which did not include any form of PR or exercise training. Feasibility was determined by uptake (≥60% of eligible participants consented) and completion (≥70% of recruited participants). Acceptability was explored by focus group discussions (FGDs) analysed thematically.

**Results:**

Seventy-nine eligible individuals (94% of screened) were referred in order to recruit 50 participants (63% uptake). The majority of participants in both intervention (72%, n=18) and control (64%, n=16) groups completed the study. Based on qualitative focus group discussions four themes emerged: (1) Increased knowledge following PR, including dispelling misbeliefs about COPD and improving medication adherence; (2) Perceived improvements in health following PR, including improved walking ability and reduced breathlessness (3) Enjoyment and benefits of cultural adaptations to PR, and (4) Challenges during PR, including adherence to exercise and travel requirements.

**Conclusion:**

Culturally adapted PR was feasible and acceptable to adults with COPD in Sri Lanka. A fully powered trial is warranted for evaluating clinical and cost-effectiveness of culturally adapted PR.

## Introduction

Accounting for 3.3 million deaths annually, chronic obstructive pulmonary disease (COPD) is ranked as the third-leading cause of death and the seventh-leading cause of poor health worldwide.^1^ The impact of COPD on individuals, families and society urges the need for evidence-based interventions to address the associated disability.^2^

PR reduces symptom burden, improves health status and quality of life of individuals living with COPD in high-income countries.^3^,^4^ Even though international guidelines (informed predominantly from evidence in high-income countries) recommend PR as an essential component of COPD management.^5–7^ There is limited evidence and clinical practice of PR in low- and middle-income countries,^8^ including Sri Lanka, which did not have a recognized PR service at the time of this trial. A non-randomised feasibility trial of PR has been reported conducted in Jaffna, Sri Lanka,^9^ and India, where peer support was employed in community-based centres.^10^ Other trials are currently being conducted in a post TB lung disease population in Uganda^11^ and Kyrgyzstan^12^ which will add to the body of knowledge previously reported in a systematic review describing low-quality evidence for PR^8^ in a low resource setting.^13^ The previously reported trial in Sri Lanka was not culturally adapted for the Sri Lankan population, which is an important consideration. Even in parts of the world where PR is routinely practiced, poor accessibility, uptake and completion remain challenging, with less than 3% of eligible patients with chronic lung disease estimated to have access to PR globally, suggesting that a one-size-fits-all approach is not appropriate for every population.^14^

Patient-centred approaches are needed to inform potential adaptations to PR, which may enhance the appeal of PR whilst still incorporating the essential components of PR.^15^ Our previous survey study revealed a clear patient preference for supervised hospital-based PR^9^ and a previously reported qualitative study proposed local adaptations for Sri Lankan adults with COPD, including nutritional supplementation with “Samaposha^®^”, singing to facilitate breathing control, the use of music in classes to boost enjoyment, and prioritising education topics of symptom management, nutritional advice, and use of medication.^16^ Accordingly, this study aimed to determine the feasibility and acceptability of a culturally adapted PR programme for adults with COPD in Sri Lanka.^17^

## Materials and Methods

### Study Design and Registration

A single-blind randomized controlled feasibility trial was conducted in adults living with COPD to compare culturally adapted PR with usual care. Ethical approval for the study was obtained from research ethics committee of Faculty of Medical Sciences, University of Sri Jayewardenepura, Sri Lanka (FMS/USJP ERC 64/19) and the University of Leicester, United Kingdom (26770). The study protocol has been published previously^17^ and the trial was prospectively registered (ISRCTN13367735). All participants provided written informed consent prior to participation.

This manuscript has been written in accordance with the Template for Intervention Description and Replication (TIDieR)^18^ (supplementary material 1) and CONsolidated Standards Of Reporting Trials (CONSORT)^19^ guidelines (supplementary material 2).

### Study Site

This study was conducted at Central Chest Clinic (CCC), Colombo, Sri Lanka, a state-owned healthcare facility that provides treatment for adults with respiratory diseases. The recruitment of participants commenced in September 2022 and closed in March 2023, with the study completed in June 2023.

### Participants

A formal sample size calculation was not performed due to the feasibility nature of the trial.^20^ Our recruitment target of 50 participants (25 in each group) is in line with previous randomized controlled PR trials for patients with COPD.^21^ Medical officers and nurses in CCC referred the adults living with COPD who were screened for eligibility. The inclusion criteria involved adults aged ≥18 years with a clinically confirmed diagnosis of COPD by a physician confirmed by spirometry based on GOLD criteria with FEV_1_/FVC<0.7, and FEV_1_<80% predicted (lung function was taken from medical records due to COVID-19 pandemic restrictions preventing spirometry being conducted as part of the trial), ≥1 exacerbation required hospitalisation in the year preceding the study, a Medical Research Council (MRC) dyspnoea grade ≥2,^22^ and able to give informed consent. Adults with comorbidities such as severe or unstable cardiovascular disease, malignant disease, locomotor difficulties, or other serious illnesses that preclude exercise were excluded from the study.

### Randomisation

An individual, independent of the research team, randomized participants (1:1) into either the intervention group or control group using Sealed Envelope.^23^

### Blinding

Due to the nature of PR, it was not possible to blind participants to their group allocation. Outcome assessors were blinded and participants were instructed not to reveal their group allocation during the discharge assessment. The blinded outcome assessors were also advised to report and document any episode of unblinding.

### Trial Intervention

#### Intervention: Culturally Adapted Pulmonary Rehabilitation

Culturally adapted PR was delivered over 6-week period, consisting of 12 sessions in total (2 sessions per week). This intervention was developed and conducted in accordance with published quality standards for PR^5^,^24^ and was delivered as face-to-face group sessions (groups consisted of 4–6 adults with COPD) by multidisciplinary team of physiotherapists, nurses and doctors. Each session lasted approximately 2 hours comprising the core elements of evidence-based rehabilitation, with 1 hour supervised exercise training, 40 minutes education session and 20 minutes group singing. All PR components followed international guidelines.^5^,^7^

Exercise training comprised warm-up exercises, aerobic training (individually prescribed walking speed and duration), strength training (upper limb exercises such as bicep curls and dumbbell upright row, and lower limb exercises such as step-ups, sitting and standing) using minimal equipment^8^ (dumbbells, plastic steps, Therabands), and warm down exercises. Walking speed was individually prescribed from the incremental shuttle walking test (ISWT), equivalent to 85% maximal oxygen capacity (VO_2_ peak). The patients were asked to walk 20 minutes at the prescribed speed, and if they were unable to continue for the entire duration, they were asked to stop to catch their breath before continuing. Over the course of the programme, participants were encouraged to aim towards walking the entire 20 minutes without stopping. Participants were asked to follow their walking exercise prescription at home on days without a scheduled PR session. Strength training was individually prescribed considering the 70% of one repetition maximum and progressed by adjusting the number of repetitions (1 to 2 sets of 8 to 12 repetitions) or the weight of the dumbbells (increasing by 0.5 kg, when the participant is able to perform 2 sets of 12 repetitions with prescribed weight) during the programme. Participants were instructed to perform one additional strength training session at home each week using minimal equipment (eg, filled water bottles as dumbbells). Education sessions were delivered by an interdisciplinary team consisting of investigators, medical officers, nurses, physiotherapists and nutritionists. Education topics included avoidance of exacerbations, chest clearance, diet, exercise, maintaining the benefit after PR, and pharmacy. All the education sessions and the instructions during PR were provided in Sinhala language as convenient for the participants.

Each group singing component consisted of a 5-minute warm-up, 10 minutes of singing followed by a 5-minute warm-down.^16^ The group singing component was specifically delivered by a trained Sri Lankan musician with experience delivering singing exercises to enhance better breathing. Participants selected songs and used the provided musical instruments to help maintain the rhythm and pace and to enhance the musical experience.

A personalised diet plan was developed by a trained, registered nutritionist (CP) for each participant. This was based on individuals’ nutritional status which was assessed prior to the beginning of PR via seven-day diet diary and body composition assessment using bio electrical impedance. A dietary supplementation, “Samaposha^®^”, consisting of a mixture of protein containing grains with similar content of nutrients of supplementation which is provided for pregnant mothers, underweight children and TB patients by the government was included as a daily component to their personalised diet plan and was monitored, via dietary logs, and adjusted accordingly throughout the intervention period.

#### Usual Care

Usual care consisted of pharmacological treatment and optimization, prescribed to patients after consultation with medical officers at the CCC. Brief information about disease conditions, medication and inhaler techniques was provided by medical officers and nurses. PR or any form of exercise training was not a part of conventional care. The participants allocated to the control group did not receive any scheduled contact, follow-up, or attention during 6-week intervention period and they were contacted after 6-weeks, only for scheduling the discharge assessment.

### Data Collection and Management

Data were collected in accordance with the recommended international dataset for PR trials across LMICs.^21^ All the scales were translated into Sinhala language, following the two-stage RECHARGE translational protocol (adapted from translation protocol for the Severe Asthma Questionnaire^25^) which included translation and back translation by two individual bilingual speakers. Collected data were entered into a Research Electronic Data Capture (REDCap) database, via a secure password protected web-interface.^21^ The records of the participants were identified by the trial identity number. Qualitative data were managed using Microsoft Word and Microsoft Excel.

### Feasibility Outcomes

The primary outcomes of the study were the feasibility of culturally adapted PR in a low-resource Sri Lankan healthcare setting. Measures to assess feasibility are outlined in detail within our previously published protocol^17^ and focused on uptake and completion. Similar to other PR feasibility trials,^26^ traffic light system progression criteria (Red-Amber-Green (RAG)) was used, where green indicated the feasibility of trial using the current methodology (recruitment ≥60%, completion ≥70%); amber indicated the need of modifications in the methodology (recruitment – 25–59%, completion – 50–69%) and red indicated non-feasibility of the trial (recruitment <25%, completion <50%),^19^,^26^,^27^ the trial was considered feasible in its current form if ≥60% of eligible participants consented to take part in the study (uptake) and if ≥70% of recruited participants randomised into the PR group attended ≥75% [9/12] sessions and the discharge assessment.

#### Acceptability

The experience of participants who completed the PR programme were explored via focus group discussions (FGDs) after their discharge assessment. These discussions mainly focused on perception of PR, perceived benefits, barriers, challenges and recommended changes to the programme. Discussions were facilitated by a trained researcher (CP) in Sinhala language, translated to English to facilitate analysis. All discussions were audio recorded (SONY ICD-UX570F recorder) with the permission of the participants. Those who did not complete PR were invited to a drop-out interview and information divulged freely by the participants was recorded as field notes.

#### Secondary Outcomes

Secondary outcomes measured before starting the trial (baseline) and after completion of the trial (discharge) are outlined in detail elsewhere.^17^ In order to facilitate the comparison across other studies and international benchmarking, selected outcomes were informed by our previous work^9^ and agreed across the wider Global RECHARGE group.^21^ Further, we used multiple questionnaires and field walking tests which are commonly used in COPD and PR literature which included: MRC dyspnoea grade, COPD Assessment Tool (CAT), Clinical COPD Questionnaire (CCQ), Work Productivity and Activity Impairment (WPAI), Euro-QOL (EQ-5D-5L), Hospital Anxiety and Depression Scale (HADS), Incremental and Endurance Shuttle Walk Tests (ISWT and ESWT), in accordance with the minimum dataset for PR in LMIC.^21^ Two ISWTs were conducted at baseline to reduce the learning effect. The minimal clinically important differences (MCID) for aforementioned outcomes were: MRC score: 1 point;^28^,^29^ CAT: 2 points;^28^ HADS: 2 points;^29^,^30^ CCQ: 0.4 points;^31^ EQ5D-5L VAS: 7 points;^32^ ISWT: 35m;^33^ ESWT: 174s;^34^ 5x sit to stand test: 1.7s.^35^

### Patient and Public Involvement (PPI)

Adults living with COPD and their family caregivers were involved in the design of the PR programme, including mode of delivery (group based, supervised PR in hospital setting), structure (biweekly 2-hour sessions) and tailoring the culturally adapted PR programme (incorporating group singing, contents of education sessions and dietary supplementation with “Samaposha^®^”) via survey^9^ and/or qualitative interviews.^16^ Informal PPI discussion then refined the implementation of new components in practice.

### Data Analysis

Of 50 randomised adults with COPD, 34 (18 participants within the PR group, and 16 in the control group) were considered for data analysis. Quantitative data were presented descriptively using IBM SPSS Statistics for Windows (version 28.0). The normality of the variables was assessed with Shapiro Wilks test. The normally distributed data were presented as means ± standard deviations and skewed data presented as median (interquartile range). No inferential statistics or imputation were conducted due to the feasibility nature of the study and the difference of pre and post mean values compared against the relevant MCIDs using descriptive statistics.

Qualitative data were analyzed using codebook thematic analysis by following six steps (i) Familiarization with the data (CP, ARJ, JM, MO); (ii) Coding the data, semantic and inductive coding approaches were adopted (CP, ARJ, JM, MO); (iii) Generating initial themes (CP, ARJ, JM, MO); (iv) Reviewing and developing themes (CP, ARJ, JM, MO, TA); (v) Refining, defining and naming themes (CP, ARJ, JM, MO, TA, AB); and (vi) Producing the report (CP, JM, MO). Microsoft Excel and Microsoft Word were used for the data management and analysis.

## Results

### Feasibility: Screening and Recruitment

**Figure 1:**
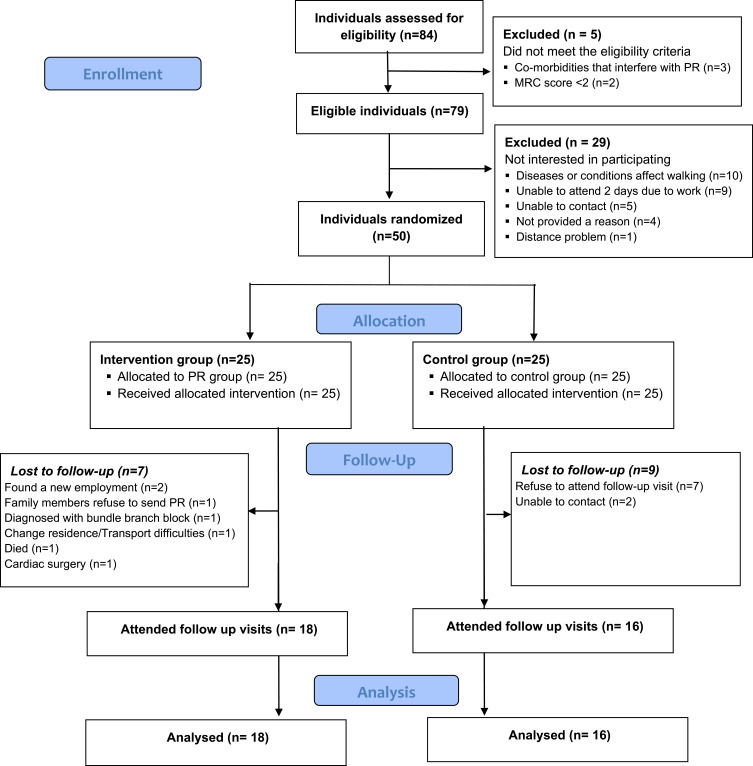
CONSORT diagram.

Eighty-four adults with COPD were screened for eligibility, of which 79 (94%) were referred to the study (Figure 1). Of these, 29 were not interested in participating, leaving 50 participants (63% of eligible) who attended the baseline assessment, consented and randomised into two groups, allocating 25 participants for each.

Seven participants allocated for the intervention group were lost to follow-up. Family members refused to send for PR (n = 1), diagnosed with bundle branch block (n = 1), found new employment (n = 2), transportation difficulties owing to change of residence (n = 1) and unrelated death of the participant (n = 1) were the causes for the dropouts from PR. Of the participants who attended all PR sessions, one underwent emergency bypass surgery and could not complete the post assessment. Nine participants allocated to the control group were refused to give an appointment for the post assessment and two were unreachable.

#### Demographic Characteristics

Intervention and control groups were generally well matched (Table 1). The majority of participants were Sinhala, Buddhist, married, and males, with a mean age of 65±8 years. One difference occurring by chance was the proportion of participants in paid work, with 8% (n = 2) of the intervention group and 48% (n = 12) of the control group; translating into the intervention group having a lower average household income.

**Table 1:** Participant Baseline Characteristics

	Group Allocation	
	Intervention (n=25)	Control (n=25)
Sex, n (%)		
*Male*	20 (80)	23 (92)
*Female*	5 (20)	2 (8)
Age at assessment (Years), mean (SD)	67±7	62±9
Age of leaving full time education (Years), median (IQR)	10 (7)	12 (4)
Educational status, n (%)		
*Never went to school*	5 (20)	2 (8)
*Up-to grade 5 (age at 10 years)*	12 (48)	14 (56)
*Up to Ordinary Level (age at 16 years)*	7 (28)	8 (32)
*Up to Advanced Level (age at 18 years)*	1 (4)	1 (4)
Civil status, n (%)		
*Single*	2 (8)	5 (20)
*Married*	23 (92)	20 (80)
Sri Lankan Ethnicity, n (%)		
*Sinhala*	15 (60)	19 (76)
*Muslim*	3 (12)	2 (8)
*Tamil*	3 (12)	3 (12)
*Other*	4 (16)	1 (4)
Religion, n (%)		
*Buddhist*	14 (56)	19 (76)
*Hindu*	3 (12)	3 (12)
*Christian*	4 (16)	1 (4)
*Islam*	4 (16)	2 (8)
Employment Status, n (%)		
*In paid work (employed)*	1 (4)	10 (40)
*In paid work (self-employed)*	1 (4)	2 (8)
*In unpaid work*	-	-
*Not in work*	23 (92)	13 (52)
Household income (monthly), n (%)		
*Bound 1 (<$36.5)*	23 (92)	13 (52)
*Bound 2 ($36.5 -$78.9)*	2 (8)	6 (24)
*Bound 3 ($79.0 -$121.4)*	-	5 (20)
*Bound 4 ($121.5 - $163.9)*	-	1 (4)
BMI (Kg/m^2^), mean (SD)	18.28±2.89	17.96±3.17
Smoking status, n (%)		
*Never*	7 (28)	2 (8)
*Current*	2 (8)	3 (12)
*Former*	16 (64)	20 (80)
Biomass fuel exposure, n (%)		
*Never*	2 (8)	3 (12)
*Current*	11 (44)	9 (36)
*Former*	12 (48)	13 (52)
Exacerbations in past 12 months, median (IQR)	2 (2)	2 (2)
Hospitalisations last 12 months, median (IQR)	1.48 (1.12)	1.80 (1.61)
Post BD FEV_1_ (n_Intervention_ =11, n_Control_ =9), mean (SD)	0.86±0.21	1.14±0.48
Post BD FVC (n_Intervention_ =11, n_Control_ =9), mean (SD)	1.90±0.49	1.98±0.66
Post BD FEV_1_/FVC Ratio (n_Intervention_ =11, n_Control_ =9), mean (SD)	0.46±0.10	0.57±0.10
Comorbidities, n (%)		
*Cardiac disease*	5 (20)	2 (8)
*Peripheral vascular disease*	1 (4)	0 (0)
*Hypertension*	5 (20)	2 (8)
*Diabetes*	4 (16)	3 (12)
*Chronic kidney disease*	0 (0)	1 (4)
*Arthritis*	1 (4)	1 (4)
Treatments, n (%)		
*LABA*	15 (60)	15 (60)
*LAMA*	1 (4)	4 (16)
*ICS/LABA*	8 (32)	8 (32)
*SABA*	21 (84)	24 (96)
*SAMA*	9 (36)	4 (16)
*Anti-histamines*	1 (4)	2 (8)
*Systemic steroids*	4 (16)	3 (12)
*Antibiotics*	1 (4)	2 (8)

**Note**: n, frequency.

**Abbreviations**: SD, standard deviation; IQR, inter quartile range; BMI, body mass index; BD, bronchodilator; FEV1, forced expiratory volume in 1 second; FVC, forced vital capacity; LABA, long acting β_2_ agonist; LAMA, long acting muscarinic antagonist; ICS, inhaled corticosteroid; SABA, short acting β_2_ agonist; SAMA short acting muscarinic antagonist.

#### Feasibility: Completion

Overall, 34 participants (68% of recruited) attended the follow-up assessment (Figure 1) with 7 participants within the PR group, and 9 in the control group being lost to follow-up. Eighteen participants (72%) in the intervention group attended 9/12 PR sessions and completed the discharge assessment (“completer”). Eighteen participants from PR group and 16 from control group were considered as intervention completers (Figure 1, Table 2).

**Table 2:** Comparison of Demographic Characteristics Between Trial Completers and Non-Completers

	Trial Completion	
	Completed (n=34)	Not Completed (n=16)
Sex, n (%)		
*Male*	28 (82)	15 (94)
*Female*	6 (18)	1 (6)
Age at assessment (Years), mean (SD),	66±7	63±10
Age of leaving full time education (Years), median (IQR)	10 (6)	12 (5)
Educational status, n (%)		
*Never went to school*	5 (15)	2 (12)
*Up-to grade 5 (age at 10 years)*	20 (20)	6 (37)
*Up to Ordinary Level (age at 16 years)*	7 (21)	8 (50)
*Up to Advanced Level (age at 18 years)*	2 (6)	-
Civil status, n (%)		
*Single*	3 (9)	4 (25)
*Married*	31 (91)	12 (75)
Sri Lankan Ethnicity, n (%)		
*Sinhala*	23 (68)	11 (69)
*Muslim*	3 (9)	2 (12)
*Tamil*	5 (15)	1 (6)
*Other*	3 (9)	2 (12)
Religion, n (%)		
*Buddhist*	22 (65)	11 (69)
*Hindu*	5 (15)	1 (6)
*Christian*	3 (9)	2 (12)
*Islam*	4 (12)	2 (12)
Employment Status, n (%)		
*In paid work (employed)*	8 (23)	3 (19)
*In paid work (self-employed)*	3 (9)	-
*In unpaid work*	-	-
*Not in work*	23 (68)	13 (81)
Household income, n (%)		
*Bound 1 (<36.5$)*	25 (73)	11 (69)
*Bound 2 (36.5$ −78.9$)*	5 (15)	3 (19)
*Bound 3 (79.0$ −121.4$)*	3 (9)	2 (12)
*Bound 4 (121.5$ - 163.9$)*	1 (3)	-
BMI (Kg/m^2^), mean (SD)	17.88±3.13	18.63±2.75
Pack years, median (IQR)	20.79 (17.08)	22.48 (14.44)
Smoking status, n (%)		
*Never*	7 (21)	1 (6)
*Current*	4 (12)	2 (12)
*Former*	23 (68)	13 (81)
Biomass fuel exposure, n (%)		
*Never*	3 (9)	2 (12)
*Current*	16 (47)	4 (25)
*Former*	15 (44)	10 (62)
Exacerbations in past 12 months, median (IQR)	2 (2)	1 (1)
Hospitalisations last 12 months, median (IQR)	1.71 (1.55)	1.50 (0.97)

**Note**: n, frequency; $, United States dollar.

**Abbreviations**: SD, standard deviation; IQR, inter quartile range.

#### Acceptability

Four FGDs were conducted among 18 participants (4–5 participants per FGD) who completed the PR programme. Four themes were generated from the data (Table 3).

**Table 3:** Generated Themes with Illustrative Quotes

Themes	Illustrative Quotes
Increased knowledge following PR	“We have been coming to the clinic for so long and have taken the medicine. But we didn’t learn anything more about the disease. I really learned a lot because of the education talks”. (P3) “Yes, learnt many unknown things from the education sessions. We do not really know about the food and drink we should take and the course of the disease. There is really no fear now. I used to feel scared when my daughter’s children came near me I worried if they would also get infected and develop a cough when they came close to me”. (P4) “But the things discussed in this are very important to us, the way we eat food without fear of food, the way we want to keep the medicine that [GP who has conduct the session] said, all of this is very important” (P11)
Perceived improvements in health following PR	“Yes, I am better than usual. I can do the [house]work more than usual. I don’t let the kids do at home because I get sick from too much work. However, when told about this, they also said that it was good. I also walk like that at home” (P3) “We really feel very good too. Therefore, we say that if possible, it would be very valuable if you could do this for all other patients”. (P8) “We really haven’t had anything like this before. We suffer a lot because of this disease. Can’t sleep, can’t eat or drink anything, can’t walk. We got a very valuable relief from this for our many problems. Actually It changed my life”. (P13) “Please, there is no any disadvantage in this programme. Everything was very good. If we were to continue like what we have done here, I feel that everything would work well”. (P9) *“Yes, I really thought that I can’t walk that fast but after coming to the programme now I can walk little faster than earlier. Really happy now” (P2)*
Enjoyment and benefits of the cultural adaptations to PR	Singing: “And we like those songs too. They are not commonly heard anymore over the radio or TV”. (P4) “I also like to sing. At home, I take the song book, listen to songs and sing. By singing songs here, I realize it is now easier to breathe and work”. (P3) “For me, I always like to sing. Earlier it was very difficult. But when I came here (to PR) I sang even with the difficulty. I feel that now it was reduced the difficulty in breathing” (P8) “There is. It is (signing component) a medicine for the mind” (P12) Dietary advice and nutritional supplementation: “I was thinking that I do not have anything to eat. Because every food I take makes me produce a lot of phlegm. Only when I ate this (Samaposha) the phlegm did not come. I feel better after taking “Samaposha” (P1) “It doesn’t cost a lot, we can’t afford food that costs a lot. Samaposha (supplementation) is affordable and it gives me strength”. (P6) “Me also now taking the supplementation from outside. It doesn’t cost a lot, we can’t do things that cost a lot” (P7)
Challenges during PR	“It was very difficult to do exercises at the beginning”. (P8) “When I get the Samaposha I have taken them with my grandchildren. They were also like it very much” (P11) “This [PR] is not available in our area hospital clinic” (P5)

##### Theme 1: Increased Knowledge Following PR

Participants expressed their positive experiences about the education component, which included improving knowledge on symptom management, improving physical activity and nutrition, controlling exacerbations and maintaining quality of life. Many participants initially had misbeliefs such as COPD spreading via droplets, which were corrected and clarified as part of the educational component of PR. With the acquired new knowledge and enhanced understanding by correcting false beliefs from the education component of PR, participants reported how they have made improvements relating to symptom self-management, more appropriate usage and better understanding of their medications, physical activity, and healthy eating following PR as their behavioral changes. These changes have made a positive impact on their lifestyle and more adherence to the PR.

##### Theme 2: Perceived Improvements in Health Following PR

Participants reported improvements in their walking ability and daily activities. Specifically, they reported an ability to walk further with reduced sensations of breathlessness following PR. They also noticed a reduction in breathing difficulty and phlegm production, contributing to an overall sense of better quality of life. Participants felt positive about continuing their PR independently beyond the intervention and were keen to recommend the PR programme to other people.

##### Theme 3: Enjoyment and Benefits of Cultural Adaptations to PR

The singing component was deemed a pleasurable activity for participants as it helped them to control their breathing while reducing their discomfort and breathlessness. Several participants talked about how they continued the signing activities outside of the scheduled PR sessions and that they will continue to do so beyond their trial participation.

Participants were highly satisfied with the supplementation and the dietary adaptations within their individualized diet plans. They reported dietary changes and such as increased intake of healthy and nutritious foods which they felt contributed to their health improvements and reduced symptom burden.

##### Theme 4: Challenges During PR

During the PR programme, there were very few barriers identified by participants. Several participants reported difficulty in adopting the exercise recommendations as they felt tired and had difficulty in moving their extremities. One participant mentioned that the provided supplementation was shared with the family members, which lowers the intake than the recommended amount. Unavailability of PR in other clinic settings was raised as a concern, particularly in relation to the travel required to attend PR.

#### Secondary Outcomes

Clinically meaningful changes were observed after PR for the MRC score, CCQ mental score, CAT score, HADS depression score, HADS anxiety score, EQ5D-5L perceived health, and ISWT distance. Improvements of the usual care group were also observed for CCQ mental score, HADS depression score, and ISWT distance (Table 4). All secondary outcome data were completed by those who completed PR, except ESWT. As per the RECHARGE protocol, two ISWTs and one ESWT were conducted at the baseline and discharge and at least 30 minutes rest between tests. A second visit was scheduled for the ESWT, which resulted in 12 participants (3 controls, 9 PR) at baseline assessment, 4 participants (4 controls, 0 PR) at discharge assessment refused to participate in ESWT.

**Table 4:** Comparison of Baseline Assessment and Post-Intervention Assessment Between Intervention Completed Group and Control Completed Group

Outcome Measure	Intervention Group (n=18)						Control Group (n=16)					
	Pre		Post		∆ Post - Pre		Pre		Post		∆ Post - Pre	
	Mean (SD)	95% CI	Mean (SD)	95% CI	∆ Mean	∆ 95% CI	Mean (SD)	95% CI	Mean (SD)	95% CI	∆ Mean	∆ 95% CI
**Health status**												
MRC score	3 (1)	3-4	2 (1)	2-2	−1.44	−1.99-−0.89	3 (1)	3-4	3 (1)	2-3	0.44	−1.05–0.18
CCQ symptom score	2.07 (0.71)	1.72–2.42	2.24 (1.25)	1.62–2.86	0.17	−0.51–0.84	2.02 (1.23)	1.36–2.67	2.17 (1.31)	1.47–2.87	0.15	−0.79–1.10
CCQ mental score	2.17 (1.03)	1.65–2.68	1.67 (1.45)	0.95–2.39	−0.5	−1.27–0.27	2.53 (1.24)	1.87–3.19	2.00 (1.98)	0.94–3.06	−0.53	−1.74–0.68
CCQ functional score	1.81 (1.18)	1.21–2.40	2.03 (1.75)	1.15–2.91	0.22	−0.48–0.92	2.38 (1.20)	1.73–3.02	3.00 (1.93)	1.96–4.04	0.62	−0.27–1.52
CCQ total score	1.97 (0.67)	1.64–2.30	2.04 (1.15)	1.47–2.61	0.07	−0.42–0.56	2.23 (1.08)	1.66–2.81	2.47 (1.31)	1.77–3.17	0.24	−0.52–0.99
CAT score	20.11 (5.37)	17.44–22.78	15.22 (6.39)	12.05–18.40	−4.89	−7.90-−1.88	22.69 (6.99)	18.96–26.41	20.94 (7.37)	17.01–24.87	−1.75	−3.90–0.40
HADS Depression score	10.39 (2.43)	9.18–11.60	5.89 (3.69)	4.05–7.73	−4.5	−6.33-−2.67	11.50 (4.21)	9.26–13.74	8.19 (3.69)	6.22–10.15	−3.31	−5.99-−0.63
HADS Anxiety score	6.33 (3.22)	4.73–7.93	4.28 (4.64)	1.97–6.58	−2.05	−4.82–0.71	7.56 (4.30)	5.27–9.86	7.50 (4.37)	5.17–9.83	−0.06	−2.04–1.92
EQ-5D-5L perceived health	50 (12)	44-56	69 (21)	59-80	19	9.36–28.97	53 (15)	45-61	57 (17)	47-66	4	−8.07–15.57
Weight	46.67 (8.18)	42.61–50.74	46.59 (8.25)	42.49–50.69	−0.08	−1.6–1.43	44.39 (10.11)	39-49.77	43.89 (9.87)	38.63–49.15	−0.5	−1.31–0.31
BMI	18.39 (3.16)	16.82–19.96	18.44 (2.85)	17.03–19.86	0.06	−0.64–0.75	17.31 (3.09)	15.66–18.96	17.00 (2.92)	15.44–18.56	−0.31	−0.63–0.01
**Exercise capacity**												
Sit to stand time for 5 repetitions	13.13 (3.43)	11.43–14.84	13.35 (2.04)	12.33–14.37	0.22	−1.65–2.09	11.64 (3.05)	10.02–13.27	12.56 (2.36)	11.30–13.81	0.92	−0.32–2.14
Distance of the best ISWT	231.11 (70.45)	196.08–266.15	286.67 (75.85)	248.95–324.39	55.56	0.02–92.92	245.63 (116.50)	183.55–307.70	291.88 (146.43)	213.85–369.90	46.25	3.98–88.52
ESWT time walked in seconds (n_Intervention_ =13, n_Control_ =9)	292.00 (125.87)	219.33–364.67	299.06 (142.64)	225.72–372.40	7.06	−134.09–104.40	305.87 (174.13)	195.24–416.50	371.46 (119.56)	299.21–443.71	65.59	−141.02–184.35

**Note**: ∆- difference.

**Abbreviations**: SD, standard deviation; CI, confident interval; BMI, body mass index; ISWT, incremental shuttle walk test; ESWT, endurance shuttle walk test; MRC, medical research council; CCQ, clinical COPD questionnaire; CAT, COPD assessment test; HADS, hospital anxiety and depression scale; EQ5D5L, euroQol five dimensions five level.

#### Adverse Events and Safety of the Intervention

The culturally adapted PR was reported as a safe intervention, evidencing no directly attributed adverse events reported during or after the PR sessions. None of the adults with COPD who completed the culturally adapted PR (n = 18) reported exacerbations or hospitalisations during the 6-week intervention period. A death was also reported following an accidental fall outside of the rehabilitation programme, which was deemed unrelated to the PR.

## Discussion

In this first trial of culturally adapted PR in Sri Lanka for adults with COPD, incorporating singing, music and personalised nutritional plan, it was feasible to recruit (63%; target ≥60%) and retain (PR: 72% and control: 64%; target ≥70%) participants. FGDs found that culturally adapted PR was acceptable for Sri Lankan adults with COPD. Participants reported enjoying the cultural components of PR (incorporating singing, nutritional supplementation and individualized diet therapy with local foods) as well as perceived improvements to their walking ability, respiratory symptoms and knowledge. Key challenges during PR related to adhering to the personalised exercise prescription and the burden of travel to attend PR classes.

Uptake in PR during the present study was greater than that observed by the only other study of PR in Sri Lanka, a single-arm non-randomised trial in Jaffna, which saw 56% of participants attend the baseline assessment.^13^ As many referred adults with COPD within this previous trial declined to participate due to lack of interest, one reason for the greater uptake in the present study may relate to PR catering to cultural diversity in its offering and delivery.^36^,^37^ When the PR is culturally adapted, participants feel familiar, comfortable and inclusive.^38^ All the adaptations of the current study were informed during the prior qualitative work among Sri Lankan adults with COPD, their caregivers and healthcare professionals.^39^ Selected adaptations were well grounded in Sri Lankan culture, ie. singing is deeply woven into Sri Lankans’ daily living, not only as a recreational activity but from lullaby to death rituals. Similar cereal supplementation to “Samaposha ^®^” is usually provided by the Sri Lankan government to every underweight child during early childhood, to every woman during the pregnancy period and even to patients with tuberculosis.

In contrast, participants feel discouraged to uptake, adhere and complete when the PR is culturally alien or unsafe^40^. A recent systematic review on self‑management interventions on COPD in LMICs in Asia supported our argument by concluding that the context-specific adaptations are mandated and the interventions should be tailored to cater to the local needs^41^. Findings from the focus group discussions also suggested that participants enjoyed the addition of a culturally adapted singing component, which likely contributed to improve participation and PR completion of Sri Lankan adults with COPD. Our culturally adapted hospital-based PR feasibility study also demonstrated superior completion rates compared to feasibility studies on PR modalities such as Community-Based PR (only 33% of recruited participants completed at least 75% of sessions and attended the post-intervention assessment).^26^ Further, a feasibility study conducted in economically deprived areas of Jodhpur in neighbouring India concluded that community-based PR was not feasible^26^ following the same RAG criteria. Interestingly, the completion rate of the current study was higher than (72% vs 43%) the non-blinded, randomised feasibility trial of an adapted PR conducted in Georgia (upper-middle-income country).^42^ The uptake and completion rates to PR within our study were similar to those reported in high-income countries. For example, according to NRAP Breathing Well Report 2024, almost a third (32%) of adults with stable COPD referred to PR started within 90 days and 68% (72% in the present study) of adults with COPD completed PR.^43^ These findings are reflective by the positive qualitative feedback from participants completing PR. Therefore, in a setting with limited prior PR exposure, it was possible to deliver a PR programme attractive to patients, with completion rates in keeping with countries with established PR services.

Although not powered to detect changes in clinical outcomes, this study found promising improvements within the PR group, surpassing published MCIDs for COPD. Specifically, these included reduced breathlessness, anxiety, and depression scores, along with improved health-related quality of life and exercise capacity. Combined with the high uptake and completion of PR as well as the positive experiences of PR attendees in the present study, our findings support and encourage further work to expand the evidence base for PR in Sri Lanka and other parts of the world without established PR programmes,^8^,^44^ including fully powered RCTs to confirm effectiveness and cost-effectiveness.

There are, however, several challenges still to be overcome. Participants who were unable to participate in the present study cited their employment responsibilities, physical inability to travel to PR appointment and already being satisfied with medical management of their COPD as key reasons not to engage with PR; in line with previous work.^13^ Due to the CCC seeing patients from across Sri Lanka, the finance cost of some patients to attend PR classes running twice weekly for six weeks (requiring unpaid leave) combined with the need to travel long distances to attend PR were particularly pertinent barriers. As the evidence base for PR grows in Sri Lanka, so will the need to establish appropriate PR centres across the country, with additional modes of PR delivery (eg, home-based programmes) to improve the accessibility of PR.^45–47^

Several limitations must be considered. Due to the nature of the PR intervention, it was not possible to blind participants to their allocation. To address this, participants were only informed of their allocation upon completion of their baseline assessment. After randomisation, a mismatch of household income pertaining to employment was observed between intervention group and control group. PR literature evinces that the financial constraints negatively impact PR participation and completion.^48^ A higher percentage of completion rate was shown in the intervention group while financial superiority was observed in the control group. Selecting a homogeneous sample^49^ (using block randomisation^50^), large enough^51^ to detect a statistically and clinically meaningful difference in outcomes between intervention and control groups, consider objectively assessable outcome measures and MCIDs validated to the relevant population, conducting a multicenter study to improve generalizability and including 6-months or 1-year post PR assessment to determine whether the acquired outcomes are maintained at a longer-term are recommended for designing a future fully powered RCT. The improvements of few health parameters were also observed in the control group; the changes in HRQOL, psychological well-being and the MRC score were of a much lower magnitude than observed in the intervention group, and consistently below the MCID unlike the intervention group. However, the results from the exercise test data were unexpected. We are confident there was no contamination of the groups, and the testers were blinded and would need larger study to understand this observation. We know from previous work that simply conducting an exercise test can improve performance but this seems an unlikely explanation.^52^

Participants were recruited solely from the CCC in the Colombo district of Sri Lanka and may not be generalisible to other parts of the country. Scalability challenges attributed to the cultural adaptations such as need of trained musicians can be prevented using recorded standard operating procedure of singing sessions or nutritional supplementation with “Samaposha^®^” can be replaced with similar locally available cereal. Suitability of proposed cultural adaptions should be explored with preliminary qualitative studies with patients and health care professionals in own precinct. However, our approach of developing a culturally appropriate PR programme can be transferred to other settings, within Sri Lanka and in parts of the world with established clinical PR services.

## Conclusion

Culturally adapted pulmonary rehabilitation using minimal equipment, including group singing and nutrition supplementation, was feasible in a low resource Sri Lankan health care setting and was acceptable for adults with COPD. The current study, as the first culturally adapted PR trial in Sri Lanka with superior uptake and completion rates, further emphasises that PR should be tailored to cater to the cultural needs, especially in LMICs with limited resources and cultural diversity.

A fully powered trial (a large-scale RCT with a homogeneous sample adequate to reliably detect a statistically and clinically meaningful difference in exercise capacity [ISWT] and psychological health [CCQ mental score, HADS], including 6-month or 1-year post PR assessment) is needed to determine the clinical and cost-effectiveness of this intervention within this context.
